# Longitudinal analysis of cerebellar volume changes across the Alzheimer's disease spectrum: implications for cognitive function and neuropsychiatric symptoms

**DOI:** 10.1002/alz70856_099275

**Published:** 2025-12-24

**Authors:** Yoo Hyun Um, Shengmin Wang, Dong Woo Kang, Sunghwan Kim, Suhyung Kim, Hyun Kook Lim

**Affiliations:** ^1^ St. Vincent' Hospital, College of Medicine, The Catholic University of Korea, Suwon, Gyeonggi‐do, Korea, Republic of (South); ^2^ College of Medicine, The Catholic University of Korea, Seoul, Korea, Republic of (South); ^3^ College of Medicine, Seoul St. Mary's Hospital, The Catholic University of Korea, Seoul, Seoul, Korea, Republic of (South); ^4^ Yeouido St. Mary's Hospital, College of Medicine, The Catholic University of Korea, Seoul, Korea, Korea, Republic of (South); ^5^ St. Vincent's Hospital, the Catholic University of Korea, Suwon, Gyeonggi‐do, Korea, Republic of (South); ^6^ The Catholic University of Korea, Seoul, SEOUL, Korea, Republic of (South)

## Abstract

**Background:**

Alzheimer's disease (AD) is a neurodegenerative disorder characterized by cognitive deficits predominantly linked to hippocampal and cortical atrophy. Emerging evidence highlights the cerebellum's involvement in both cognitive and neuropsychiatric symptoms of AD, challenging its traditional characterization as resistant to AD pathology. This study investigates longitudinal cerebellar volume changes across the AD spectrum, from preclinical stages to dementia, and their associations with cognitive and neuropsychiatric symptoms.

**Methods:**

A total of 229 older adults from the Catholic Aging Brain Imaging Database (CABID) were classified into four groups: Normal Cognition (NC), Preclinical AD (PAD), Aβ‐positive Mild Cognitive Impairment (MCI), and AD Dementia (ADD). Over an average follow‐up of two years, participants underwent MRI, PET imaging, and clinical assessments. Regional cerebellar volumes were analyzed using SPM12 with the SUIT atlas, and statistical models assessed group differences and longitudinal changes.

**Results:**

At baseline, significant cerebellar volume reductions were identified in the ADD group, particularly in Crus I, Crus II, and the Vermis. Longitudinal analyses revealed progressive volume reductions in the PAD group, notably in the left Crus I and left VI, preceding significant cognitive decline. In contrast, no longitudinal changes were observed in the NC or MCI groups. Notably, cerebellar volume reductions in the PAD group correlated with episodic memory performance, while reductions in the ADD group were linked to neuropsychiatric symptoms, including psychosis, irritability, and euphoria. These findings underscore the cerebellum's involvement in AD pathology and its interconnected role with cortical regions in executive function and memory.

**Conclusion:**

Longitudinal cerebellar volume changes offer critical insights into the trajectory of AD, suggesting that the cerebellum may serve as an early biomarker and potential therapeutic target in AD progression.

